# Advancements in hydrogel‐based embolic agents: Categorized by therapeutic mechanisms

**DOI:** 10.1002/cam4.70183

**Published:** 2024-10-23

**Authors:** Shenbo Zhang, Rui Lv, Zhe Zhang, Zhiwei Wang, Zhengyu Jin

**Affiliations:** ^1^ Department of Radiology, Peking Union Medical College Hospital Chinese Academy of Medical Sciences Beijing China

**Keywords:** embolic agents, hydrogels, instrumentation, interventional radiology, therapeutic embolization

## Abstract

**Background:**

Transcatheter arterial embolization (TAE) is a crucial technique in interventional radiology. Hydrogel‐based embolic agents show promise due to their phase transition and drug‐loading capabilities. However, existing categorizations of these agents are confusing.

**Aims:**

This review tackles the challenge of categorizing hydrogel‐based embolic agents based on their therapeutic mechanisms, including transportation, accumulation, interaction, and elimination. It also addresses current challenges and controversies in the field while highlighting future directions for hydrogel‐based embolicagents.

**Materials and Methods:**

We conducted a systematic review of papers published in PUBMED from 2004 to 2024, focusing primarily on preclinical trials.

**Results:**

Various kinds of hydrogel embolic agents were introduced according to their therapeutic mechanisms.

**Discussion:**

Most hydrogel embolic agents were specifically designed for effective accumulation and interaction. Recent advancement highlight the potential of multifunctional hydrogel embolic agents.

**Conclusion:**

This new categorizations provided valuable insights into hydrogel embolic agents, potentially guiding material scientists and interventional radiologists in the development of novel hydrogel embolic agents in transarterial embolization.

## Graphical abstract

Transcatheter arterial embolization is an promising technique in treating a variety of diseases, including cancers. However, the previous category did not fix the multifunctional therapeutic agents‐loaded hydrogel embolic agents. In our comprehensive review, we present a fresh perspective on the in vivo procedure of transcatheter arterial embolization, including transition, accumulation, interaction, and elimination. Our aim is to inspire material scientists and interventional radiologists to explore innovative perspectives and approaches in this field.

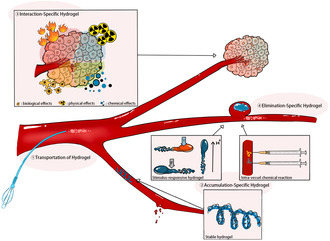

## 1 INTRODUCTION

Transcatheter arterial embolization (TAE) is pivotal in interventional radiology, delivering embolic agents to target vessels through catheter‐based interventions, inducing therapeutic effects by altering hemodynamics and diminishing blood supply. There have been substantial improvements in understanding, equipment, and application in TAE since the 1960s.^1^, ^2^ Notably, transcatheter arterial chemoembolization (TACE) introduced by Yamada et al. in 1983 incorporated antineoplastic agents during embolization, expanding treatment options.^3^ Advancements in imaging technologies, medical devices, contrast agents, and embolic agents have broadened procedures available to interventional radiologists, including alleviating benign chronic inflammatory joint pain.^2^, ^4^ However, the evolution of embolic agents, particularly hydrogel‐based ones, poses challenges in systematic classification.

The introduction of hydrogels by Wichterle and Lim in 1960 revolutionized biomedical materials, offering versatile three‐dimensional hydrophilic polymer networks capable of absorbing water and solutes. Hydrogels have diverse applications in biomedicine, from contact lenses to drug delivery, wound dressings, tissue engineering, hygiene products, and embolic agents.^5^, ^6^, ^7^, ^8^, ^9^ Hydrogels exhibit diverse physical properties depending on the combinations between their molecules, ranging from stable to reversible. Additionally, hydrogels are heterogeneous, containing pores of various sizes capable of loading solutions and cells.^5^, ^6^, ^9^ Despite attempts to categorize hydrogel embolic agents, existing classifications remain confusing in bridging material science and clinical demand.^6^, ^7^, ^8^

This review grapples with the challenge of categorizing hydrogel‐based embolic agents in the context of their therapeutic mechanisms, encompassing transportation, accumulation, interaction, and elimination. Transportation involves two stages: intra‐catheter transportation, which refers to the motion within the catheter, and intra‐vessel transportation, which describes the expulsion from the catheter tip into the vessel and its subsequent movement to the target site. Accumulation, a critical status in embolization, denotes the aggregation of embolic agents within vessels, leading to occlusion of blood flow. Interaction encompasses various effects between embolic agents and surrounding tissues, except for embolization effects, such as those inducing hyperthermia or cell transplantation. Elimination refers to the removal of embolic agents from their accumulated sites.

**FIGURE 1:**
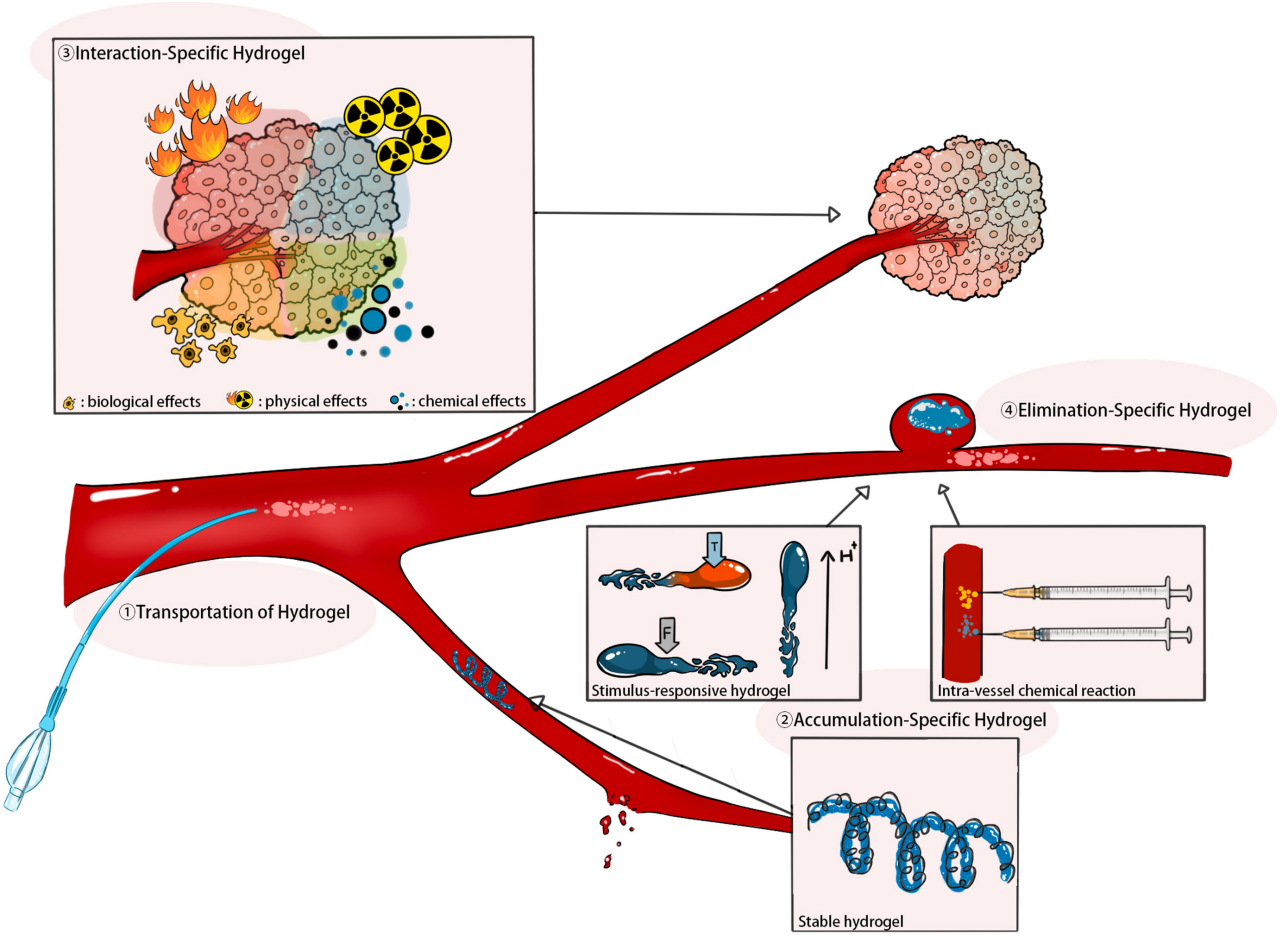
Illustration of transportation, accumulation, interaction, and elimination.

This manuscript provides an introduction to the latest hydrogel‐based embolic agents, focusing on innovations in transportation, accumulation, interaction, and elimination (Figure 1). Additionally, it addresses current challenges and controversies within the field, aiming to inspire material scientists and interventional radiologists to explore novel perspectives on hydrogel embolic agents in TAE.

## 2 HYDROGEL OF DIFFERENT THERAPEUTIC MECHANISMS

Although every embolic agent will undergo transportation, accumulation, interaction, and elimination in vivo, each phase is influenced by the unique properties of the materials. While the subtitles in this section denote the primary purpose of hydrogel embolic agents, some are multifunctional, as detailed in Table 1.

**TABLE 1:** Summary of hydrogel embolic agent.

Year	Author	Materials	Accumulation	Interaction	Elimination	Experimental subjects	Study purpose
2021^13^	Hongo N. et al.	AZUR (hydrogel‐coated coil)	Stable hydrogel	‐	‐	Patients	Comparison between 38 and 39 vessel disease patients who underwent bare coil and AZUR coil embolization
2015^32^	Jung S. C. et al.	Bilayered polyvinyl alcohol strand	Stable hydrogel	‐	‐	Canine	Aneurysm model on canines
2006^11^	Bui, J. T et al.	Hydrocoil®	Stable hydrogel	‐	‐	Patients	3 patients with mesenteric hemorrhage
2008^87^	Plenk, H. Jr et al.	HydroCoil®	Stable hydrogel	Biological effects (cartilage neoformation)	‐	Rabbit	Artificial aneurysm on rabbits
2012^31^	Khan S.‐N. H. et al.	HydroCoil®	Stable hydrogel		‐	Patients	Comparison between hydrocoil and bare coil in Intracranial aneurysms patients
2013^88^	Lopez‐Benitez R. et al.	Hydrocoil®	Stable hydrogel	‐	‐	Patients	Embolize narrow vessel in 3 patients
2018^30^	Fohlen A. et al.	HydroCoil®	Stable hydrogel	‐	‐	Sheep	Mechanism verification
2021^89^	Healan S. J et al.	HydroCoil®	Stable hydrogel	‐	‐	Patients	Case report of aneurysm and coronary artery fistular
2010^34^	Killer M. et al.	HydroFill® (MicroVention Terumo) and HydroSoft® (MicroVention Terumo)	Stable hydrogel	Biological effects	‐	Rabbit	Bifurcation aneurysms model on rabbits
2010^73^	Reinges M. H. T. et al.	HydroCoil®	Stable hydrogel	Biological effects	‐	Rabbit	Artificial aneurysm on rabbits
2011^33^	Guimaraes M. et al.	Nonmetallic injectable hydrogel (63% barium sulfate, 25% poly (ethylene glycol), 10% diacrylamide, 11% sodium acrylate, and 1% *N*, *N*‐methyl enebisacrylamide)	Stable hydrogel	‐	‐	Pig	Mechanism verification
2018^12^	Zhang Y et al.	One‐step copolymerization of acrylonitrile (AN, dipole monomer), acrylamide (AAm, hydrogen‐bonding monomer), and a long flexible PEG3kDMA crosslinker	Stable hydrogel	‐	‐	Pig	Mechanism verification
2023^90^	Zhou H. et al.	(*N*‐isopropyl acrylamide)‐co‐acrylic acid nanogel (NIPAM‐co‐AA)	Temperature‐responsive	‐	‐	Rabbit	Mechanism verification
2023^79^	Chen C et al.	Chitosan/sodium alginate microsphere dispersed in chitosan/sodium glycerophate hydrogel	Temperature‐responsive	Chemical effects (drug release)	Degradable (degradation rate of 58.869 ± 1.754% in 4 weeks)	‐	In vitro only
2016^45^	A. et al.	Doxorubicin and sorafenib loaded silk‐elastin‐like protein polymer	Temperature‐responsive	Chemical effects	‐	‐	In vitro only
2015^39^	Qian K. et al.	Doxorubicin‐loaded p(*N*‐isopropylacrylamide‐co‐butyl methylacrylate) nanogels–iohexol dispersions (IBi‐D)	Temperature‐responsive	Chemical effects	‐	Rabbit	VX2 liver cancer model on rabbits
2014^91^	Chen X. et al.	Grafting poly(*N*‐isopropylacrylamide) chains on bacterial cellulose nanowhiskers	Temperature‐responsive	‐	‐	‐	In vitro only
2015^44^	Poursaid A. et al.	In situ gelling silk‐elastin‐like protein polymer (SELP)	Temperature‐responsive	‐	‐	Rabbit	Mechanism verification
2019^51^	Yang H. et al.	Methoxy PEG‐poly(D,L‐lactide) copolymer (mPEG‐PLA) thermogel	Temperature‐responsive	‐	Recanalization in 1 h	Pig	Mechanism verification
2011^24^	Zhao Y. et al.	p(N‐isopropylacrylamide‐co‐butyl methylacrylate) (PIB) nanohydrogel	Temperature‐responsive	‐	‐	Rabbit	VX2 liver cancer model on rabbits
2020^53^	He Y. et al.	Pluronic F127 (F127) and hydroxymethyl cellulose (HPMC) and Iohexol powder	Temperature‐responsive	Chemical effects (DOX loaded)	‐	Canine and rabbit	Healthy canine and VX2 liver cancer model on rabbits
2021^25^	Wang Q. et al.	Poloxamer 407 (F127)/hydroxymethyl cellulose (HPMC)/sodium alginate (SA)‐derived hydrogel (FHSgel)	Temperature‐responsive	‐	‐	Rabbit	VX2 liver cancer model on rabbits
2016^52^	Huang L. et al.	Poloxamer 407, sodium alginate, hydroxymethyl cellulose and iodixanol (PSHI), together with Ca2+ (PSHI‐Ca2+)	Temperature‐responsive	‐	Slow erosion rate	Rabbit	VX2 liver cancer model on rabbits
2022^92^	Hatlevik O. et al.	Silk‐elastin‐like protein polymer	Temperature‐responsive	‐	Degradable	Pig	Mechanism verification
2023^93^	Zhao W. et al.	Stably distributing ethiodized poppy seed oil and epirubicin in the blend hydrogel of methylcellulose and xanthan gum	Temperature‐responsive	Chemical effects	‐	Rabbit	Ear‐loaded VX2 rabbit
2011^48^	Wang Y. et al.	Thermosensitive chitosan/glycerophosphate (C/GP) hydrogel	Temperature‐responsive	‐	‐	Rabbit	Mechanism verification
2014^70^	Lee C.‐M. et al.	Chitosan micro‐hydrogels (CMH)	‐	‐	‐	Rat	Mechanism verification
2015^49^	Salis A. et al.	Thermosensitive chitosan/glycerophosphate (C/GP) hydrogel	Temperature‐responsive	‐	‐	Ex vivo bovine liver	Mechanism verification
2021^94^	Fujiwara S. et al.	Triblock copolymers of aliphatic polyester and poly(ethylene glycol) and its derivative containing acrylate group at the termini mixed with aldehyde group‐capped Pluronic P‐123 (PL‐CHO)	Temperature‐responsive	‐	Degradable	Ex vivo rat aorta	Mechanism verification
2022^50^	Yan X. et al.	Triblock polymer matrix and reduced graphene oxide nanosheets decorated with iron oxide nanoparticles (Fe3O4@rGO, denoted as FG)	Temperature‐responsive	Physical effects (magnetic hyperthermia)	‐	Rabbit	VX2 liver cancer model on rabbits
2020^77^	Hu J. et al.	Decellularized cardiac extracellular matrix‐based nanocomposite hydrogel	Temperature‐responsive and shear‐thinning	Biological effects (antimicrobial and proregenerative)	Recanalizing in day 14	Pig	Mechanism verification
2018^95^	Hwang H. et al.	Chitosan‐based hydrogel microparticles	‐	‐	‐	Rabbit	VX2 liver cancer model on rabbits
2012^72^	Chabrot P. et al.	Chitosan/glycerophosphate hydrogel loaded with a sclerosant (sodium tetradecyl sulfate, STS)	‐	Chemical effects	‐	Rabbit	Mechanism verification
2017^68^	Hwang H. et al.	^131^I‐labeled chitosan hydrogels particle	‐	Physical effects	‐	Rat	VX2 liver cancer model on rabbits
2014^70^	Lee C.‐M. et al.	Doxorubicin‐loaded I‐131‐labeled chitosan microhydrogels (I‐131‐ CMH)	‐	Physical effects and chemical effects	‐	Rat	MDA‐MB231 tumor model on rats
2018^74^	Touma J et al.	Bone marrow derived mesenchymatous stem cells (BM‐MSCs) hyaluronic acid hydrogel	‐	Biological effects (induce accumulation of collagen and smooth muscle cells in embolized vessel)	‐	Pig	AVM model on pigs
2023^75^	Lv P. et al.	Calcium alginate hydrogel microsphere with oncolytic adenoviruses (OA) encapsulated	‐	Biological effects (oncolytic)	‐	Rabbit	VX2 liver cancer model on rabbits
2018^76^	Baba Y. et al.	PuraMatrix (16 peptide)	‐	Biological effects	‐	Pig	Mechanism verification
2016^78^	Zhou F. et al.	Graphene‐oxide contained generation five poly (amidoamine) dendrimers	‐	‐	Slow erosion rate	Rabbit	Mechanism verification

*Note*: Transportation is not included in this table, as most hydrogel embolic agents do not have specific designs for transportation.

### 2.1 Transportation of hydrogel

The successful delivery of embolic agents to target vessels hinges on factors like catheter navigability and hydrogel viscosity. Injection force, crucial for smooth delivery, falls below 50 N for ideal injectability, with up to 100 N still feasible but challenging. Viscosity, below 150 CP, ensures smooth injection. Yet, few embolic agents are tailored specifically for transportation, particularly in flexible and flowable hydrogel forms.^10^

#### 2.1.1 Intra‐catheter transportation

Most hydrogel embolic agents are delivered using standard catheters or micro‐catheters, making them compatible with existing interventional procedures,^11^, ^12^, ^13^ An exception is the Embrace™ Hydrogel Embolic System, which features a specialized dual‐lumen catheter for separately transporting the polymer precursor and initiator precursor.^14^ This system suggests that advancements in hydrogel embolic agent transportation could be highly promising. However, it is crucial to assess their compatibility with current interventional devices.

#### 2.1.2 Intra‐vessel transportation

Navigating vessels presents challenges due to their heterogeneity in branch, shape, and caliber.^15^, ^16^ Non‐targeted embolization is a potential complication of transarterial embolization (TAE), where embolic agents inadvertently enter incorrect vessels and obstruct the blood supply to healthy tissue. This highlights the critical importance of precise intra‐vessel transportation.^17^, ^18^ Dual‐lumen balloon catheter‐assisted embolization has been shown to mitigate non‐targeted embolization in TAE when using fluid embolic agents and coils.^19^, ^20^, ^21^, ^22^ However, research on dual‐lumen balloon‐assisted hydrogel embolization is limited, as most hydrogel embolic agents remain in the preclinical stage. Jerry C Ku et al. reported using a photosensitive hydrogel‐based embolic agent with balloon assistance to embolize wide‐necked aneurysms in pigs, have shown promise in minimizing non‐target embolization.^23^

In summary, most hydrogel embolic agents prioritize accumulation over transportation. The next section delves into specific strategies to achieve precise accumulation.

### 2.2 Accumulation‐specific hydrogel

Efficient accumulation not only ensures robust embolization but also reduces non‐targeted embolization risks. Robust embolization entails firm occlusion and proper distribution within vessels.^24^, ^25^ Non‐targeted embolization risks include downstream and reflux embolization.^26^, ^27^, ^28^ Methods for controlled accumulation include stable hydrogel formations (e.g., microspheres or coils), stimulus‐reactive hydrogels, and intra‐vessel chemical reactions. The diverse physical properties of hydrogels, ranging from stable to reversible, enable tailored strategies for precise accumulation. Stable hydrogels ensure proper occlusion, while reversible hydrogels respond to specific triggers, facilitating controlled embolization.

#### 2.2.1 Stable hydrogel

**FIGURE 2:**
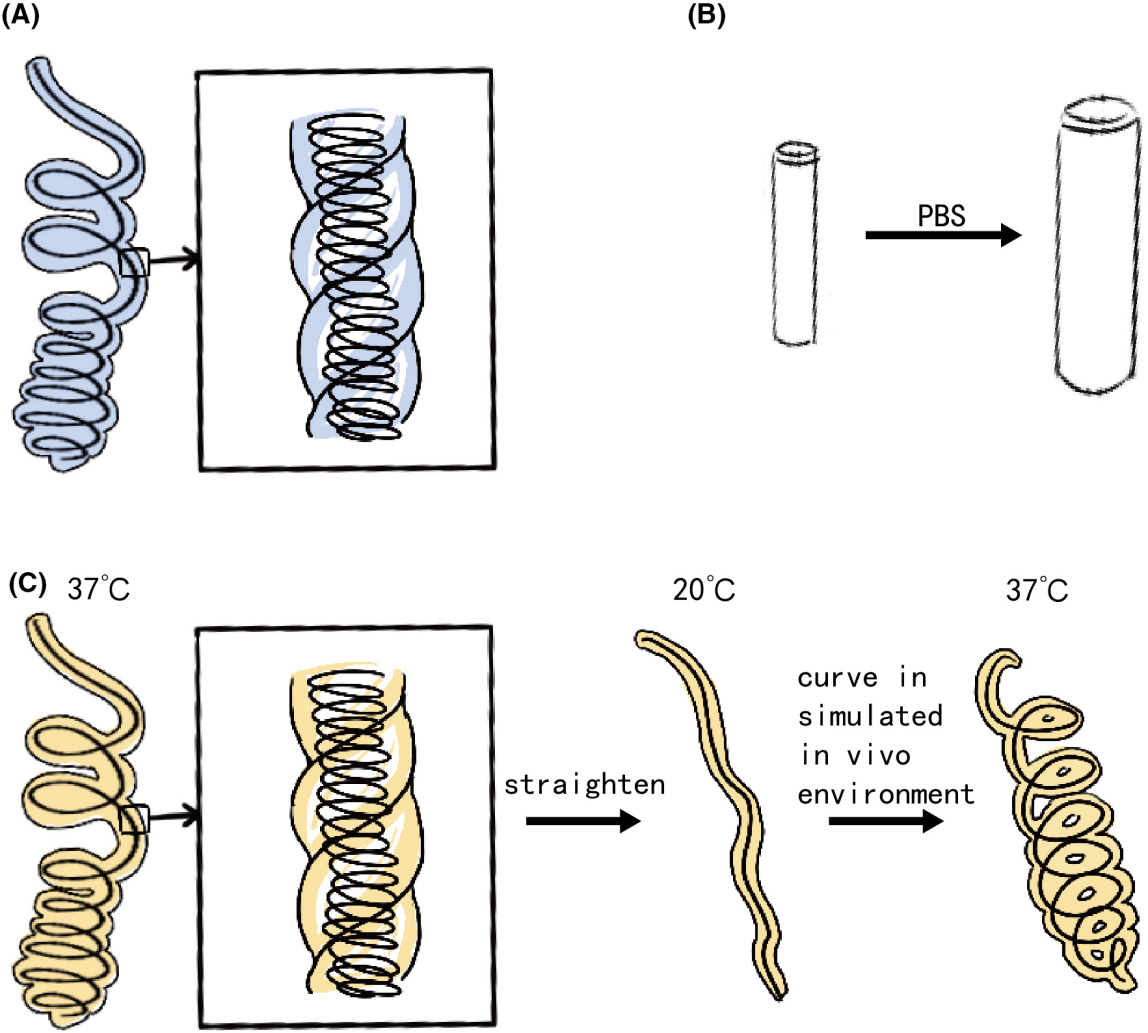
Stable hydrogels (A) HydroCoil®; (B) bilayered polyvinyl alcohol hydrogel coils; (C) shape memory hydrogel coil.

In recent years, HydroCoil™, a hydrogel‐coated metallic coil, has garnered significant attention in animal and patient studies^11^, ^29^ (Figure 2A). The hydrogel coating reduces the thrombus occlusion ratio of the bare coil, potentially lowering the recanalization rate.^30^, ^31^ Additionally, the swelling properties of hydrogel improve its volumetric filling capacity.^13^ Norio Hongo et al. observed shorter embolization segments and coil lengths with HydroCoil™ compared to bare coil embolization in vessel disease patients,^13^ while the embolic efficacy was the same.

Beyond HydroCoil®, novel hydrogel coils have emerged. Jung Seung Chai et al. investigated bilayered polyvinyl alcohol hydrogel coils in canine models (Figure 2B), showing comparable packing density to metallic coils. However, three in four aneurysm model canines were observed recanalization 4 weeks after embolization.^32^ Marcelo Guimaraes et al. tested a nonmetallic injectable coil hydrogel on healthy pigs, demonstrating similar embolization effects to HydroCoil®.^33^ Yinyu Zhang et al. introduced a shape memory hydrogel coil. The hydrogel softens at body temperature and stiffens at room temperature. To impart the coil shape memory property, the hydrogel was twisted on a steel coil, and then the coil was straightened at body temperature and cooled down to room temperature (Figure 2C). Initial trials on healthy pigs have verified the embolization efficiency of the coil.^12^

However, the hydrogel coils share some common limitations. First, the hydrogel may tear off from the coil and lead to non‐targeted embolization. Second, the hydrogel coil is stiffer than the bare coil, which may cause the movement of the catheter tip. Third, the hydrogel coil may swell in the catheter and lead to occlusion of the catheter.^12^, ^13^, ^31^ New construction of the hydrogel coils and softer hydrogel was reported to solve the second limitation.^34^ Additionally, techniques like employing a one‐way stopcock between the catheter hub and touhy can mitigate risks associated with HydroCoil® use.^31^

#### 2.2.2 Stimulus‐responsive hydrogel

Smooth transportation and robust accumulation require contrary physical properties of the hydrogel. For proper accumulation, a viscosity range from 30 to 150 CP is considered suitable for small capillaries, high viscosity of more than 400 CP is preferred for large cavities like aneurysms.^10^ Except for using stable hydrogel to get it stuck at the target vessels, making use of the specific environment at the target vessels to stimulate the sol–gel transition of hydrogel offers another avenue. Additionally, to prevent venous washout, which may lead to downstream embolization, the ideal sol–gel transition in arterial vessels is less than 5 min.^10^

**FIGURE 3:**
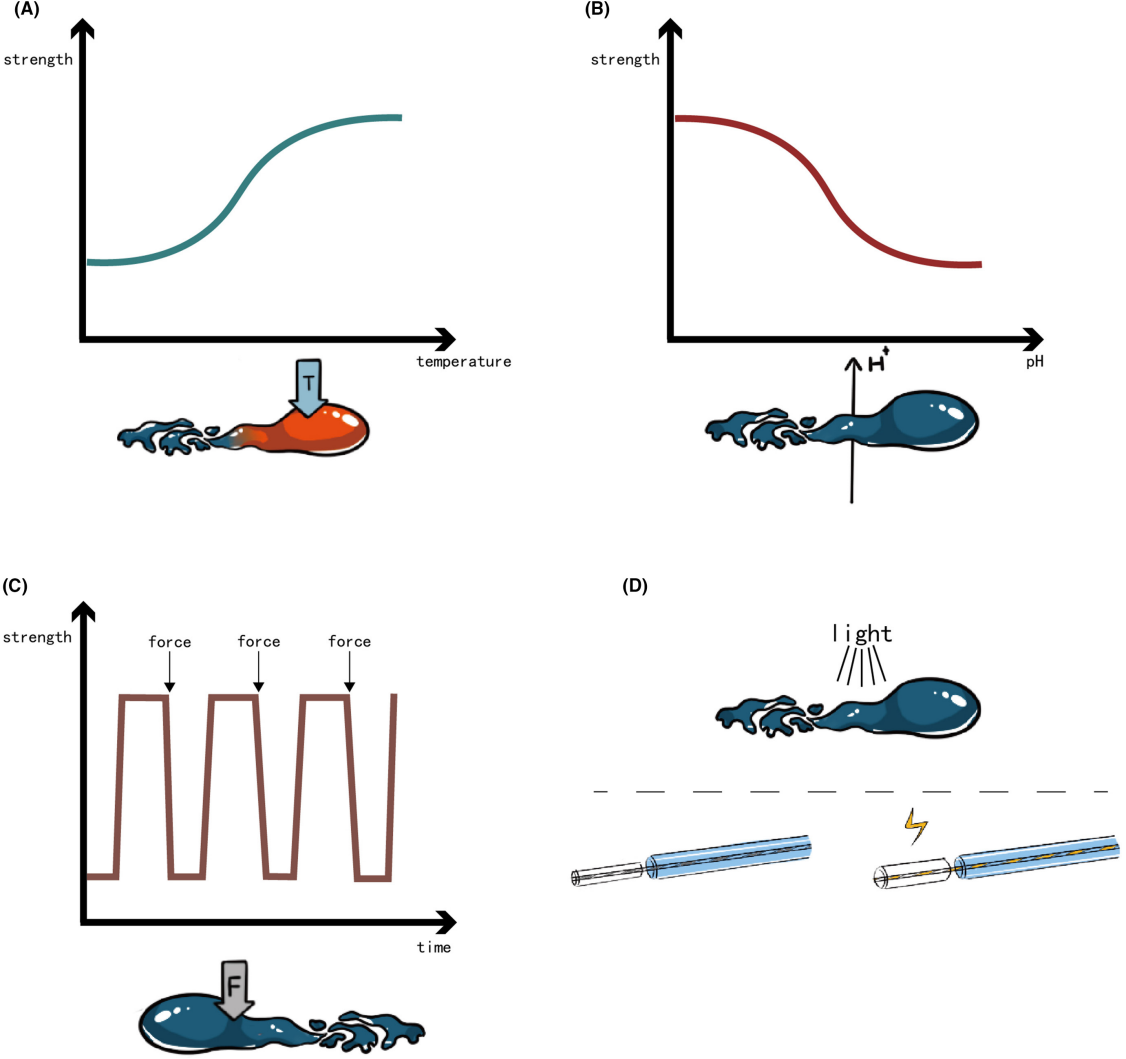
Stimulus‐responsive hydrogel: (A) Illustration of temperature‐responsive hydrogels; (B) illustration of pH‐responsive hydrogels; (C) illustration of shear‐thinning hydrogels; (D) illustration of electro‐responsive and photo‐responsive hydrogels.

Temperature‐responsive hydrogels offer a solution in embolization procedures, which convert from solution to gel phase at about body temperature. The transition temperature, termed the lower critical solution temperature (LCST), plays a pivotal role in their functionality^35^ (Figure 3A).

Poly(N‐isopropylacrylamide) (PNIPAM), a temperature‐sensitive polymer, exhibits a sol–gel transition at 32°C, making it ideal for embolization applications.^24^, ^36^, ^37^, ^38^, ^39^ Yanbing Zhao et al. synthesized a nanohydrogel dispersed with iohexol for X‐ray visibility, showcasing a tuned LCST of 36.5°C. In vitro experiments revealed varying gelation times at different initial temperatures, while in vivo studies on VX2 liver tumor‐bearing rabbits demonstrated efficient embolization.^24^ Kun Qian et al. enhanced the nanohydrogel by loading doxorubicin, with an LCST around 33°C. Interestingly, the viscosity of hydrogel initially increased and then decreased with higher doxorubicin concentrations. The doxorubicin‐loaded hydrogel embolization group exhibited significantly lower tumor growth rates compared to infusion alone.^39^ Ling Li et al introduced an X‐ray and ultrasound visible temperature‐responsive hydrogel. The gelation temperature was about 37°C. Embolization in VX2 liver tumor‐bearing rabbits demonstrated robust efficiency and sustained ultrasound visibility over 4 weeks.^36^ PNIPAM was also added to other materials without temperature responding to generate temperature‐responsive hydrogel. Zheng et al. grafted PNIPAM to lignin via atom transfer radical polymerization to synthesize a temperature temperature‐responsive poly(N‐isopropylamide)‐grafted lignin hydrogel. The hydrogel showed low shear viscosity (44CP) at room temperature and high strength (G' is 3099 Pa) at body temperature. In vivo testing on kidneys of healthy rabbits revealed evident hydrogel filling in kidney vessels 12 weeks post‐embolization.^40^

Silk‐Elastin‐like Protein Polymer (SELP) is another promising temperature‐responsive hydrogel embolic agent. Different from the PNIPAM, SELP is a product of genetic engineering.^41^, ^42^, ^43^ The monomer of SELP consists of elastin‐like and silk‐like polypeptide sequences. Its temperature‐sensitive polymerization involves two key steps: elastin‐like domains trigger a rapid phase transition above a specific temperature, followed by silk‐like domains inducing slow β‐sheet formation to counter phase separation.^35^ Azadeh Poursaid et al. crafted SELP hydrogel SELP‐815 K, formulated at 12% w/w through shear processing. This hydrogel exhibited an optimal viscosity of around 100 CP at room temperature, with a gelation time of 3.5 minutes and remarkable stiffness (4.4E5 Pa). In a rabbit liver embolization experiment, SELP‐815 K effectively occluded the terminal hepatic artery without downstream embolization evidenced histologically.^44^ Poursaid, A. et al. further explored SELP by loading doxorubicin and sorafenib, observing minimal influence on hydrogel viscosity and stiffness, albeit with prolonged gelation time (over 5 min).^45^

Chitosan, a biocompatible and biodegradable polymer derived from chitin deacetylation,^46^ has gained attention for its sol–gel transition with β‐glycerophosphate (C/GP) at body temperature.^47^ Wang et al. conducted a preliminary trial of C/GP on healthy rabbit kidneys, resulting in complete kidney atrophy confirmed both macroscopically and histologically after 8 weeks.^48^ Andrea Salis et al. further improved the C/GP by loading indocyanine green to it. The capacity to absorb and emit in the near‐infrared spectral range of indocyanine green imparted the hydrogel a potential to realize intraoperative fluorescence imaging. The gelation time changed with the concentration of chitosan and β‐glycerophosphate. Optimal rheological characterization was observed on C1.6/GP18d, gelling in 2 min and demonstrating shear‐thinning behavior conducive to controlled accumulation. The embolization trial on ex vivo bovine liver with the visualization of Photo Dynamic Eye that can detect near‐infrared fluorescence showed a uniform distributed fluorescence at the embolization site.^49^

Attempts of other temperature‐responsive materials as embolic agents presented promising results. Xu Yan et al. explored a temperature‐responsive triblock polymer matrix integrated with reduced graphene oxide nanosheets and iron oxide nanoparticles (Fe_3_O_4_@rGO) for embolizing the feeding artery in a rabbit VX2 liver tumor model. This hydrogel demonstrated excellent injectability with an inject force of about 2 N in a 29G catheter. However, its gelation temperature of approximately 14°C limits its room temperature storage capability. Notably, the magnetic hyperthermia property of hydrogel will be discussed in the subsequent section on “interaction‐specific hydrogels.”^50^ Hantao Yanga et al. introduced a reversible temperature‐responsive hydrogel using methoxy PEG‐poly(D, L‐lactide) copolymer, which gelled at around 30°C. In vivo embolization on the porcine pharyngeal artery and its branches demonstrated complete occlusion of the target vessels, although recanalization occurred one hour post‐embolization.^51^ Poloxamer 407, also known as Pluronic F127, was employed to confer thermal response properties to hydrogels. Various formulations, including poloxamer 407/hydroxymethyl cellulose (HPMC)/sodium alginate (SA)‐derived hydrogel, Pluronic F127 and HPMC with Iohexol powder, and poloxamer 407/SA/HPMC/iodixanol/Ca^2+^ hydrogel, were tested in vitro and in vivo (animal experiments).^25^, ^52^, ^53^

Despite the rapid advancements in temperature‐responsive hydrogel embolic agents, further investigation into precise control of hydrogel accumulation is warranted. Factors such as patient body temperature, catheter length in a vessel, viscosity change rate during gelation, and injection force can influence hydrogel accumulation. Moreover, improper gelation in the catheter may lead to catheter obstruction.

pH‐responsive hydrogels make use of the pH value at embolization site, altering polymer chain charges from the catheter to the target vessel^54^ (Figure 3B). Hwang et al. synthesized a biodegradable pH‐responsive chitosan hydrogel Janus particles. The particles own zwitterionic nature, which provided the hydrogel with a pH‐dependent ionic interaction. CT scans of healthy rabbit kidneys post‐embolization showcased even particle accumulation at the target site.^55^ Another pH‐responsive hydrogel adapted to both physiological (pH 7.4) and tumor (pH 6.5–7.0) conditions, comprising poly(e‐caprolactone), sulfamethazine, and poly(ethylene glycol). It transited from a liquid phase at high pH (8.0) to a gel phase at low pH (7.4 or less), with viscosity ranging from 1 Pa·s to nearly 10,000 Pa·s during gelation. The short‐term embolization efficiency was confirmed on both CT scans and histologic slides (5 h after embolization) of the rabbit VX2 liver tumor model.^56^ Interestingly, Dedai Lu et al. introduced a noninvasive pH‐responsive hydrogel based on acidic microenvironment‐responsive doxorubicin‐loaded hyperbranched poly[l‐threonine‐b‐(l‐glutamic‐ran‐l‐tyrosine)]s (HPTTG). The optimized ratio of l‐Tyr:l‐Glu was 58:18, exhibiting a sol–gel transition pH of 6.8. The embolization efficacy was demonstrated on subcutaneous tumor‐bearing rats and VX2 liver tumor‐bearing rabbits. The HPTTG‐injected groups exhibited prolonged survival. The in vivo tracing of the labeled HPTTG showed a high concentration at the tumor site and low at other regions of rats and rabbits. Digital subtraction angiography (DSA) images of the rabbit hepatic tumor displayed significant blood flow blockage 8 and 12 h after injection of HPTTG.^57^ PH‐responsive hydrogels addressed the limitation of temperature‐responsive hydrogels that its phase transition occurs in the catheter. However, further investigation into the precise accumulation of pH‐responsive hydrogels is still required.

Shear‐thinning hydrogels undergo reversible sol–gel transitions under mechanical shake or shear stress, quickly reforming networks post‐external force removal^58^, ^59^ (Figure 3C). Jingjie Hu et al. deeply investigated a decellularized‐cardiac‐extracellular‐matrix‐based hydrogel with shear‐thinning properties. The injection force of the hydrogel measured was less than 25 N, which indicated an easy injection. Histological slides of embolized rabbit kidneys showed the hydrogel in blood vessels with a diameter of 200 μm.^60^

Various other stimuli‐responsive hydrogels exist, including electro‐responsive and photo‐responsive types (Figure 3D). Verbrugghe et al. presented a Pluronic methacrylic acid hydrogel with strong electro‐mechanical actuation, enabling significant volume contraction and expansion. This characteristic holds promise for treating large vessel diseases like aneurysms.^61^ Dobashi et al. introduced a photo‐responsive poly(ethylene glycol diacrylate)‐nanosilicate hydrogel, exhibiting increased viscosity and storage modulus post‐ultraviolet irradiation. It was transported with a specific fiber‐optical catheter. Preliminary trials on health, arteriovenous malformations, vascular injuries, and aneurysm model pigs demonstrated its therapeutic efficacy.^62^

#### 2.2.3 Intra‐vessel chemical reaction

**FIGURE 4:**
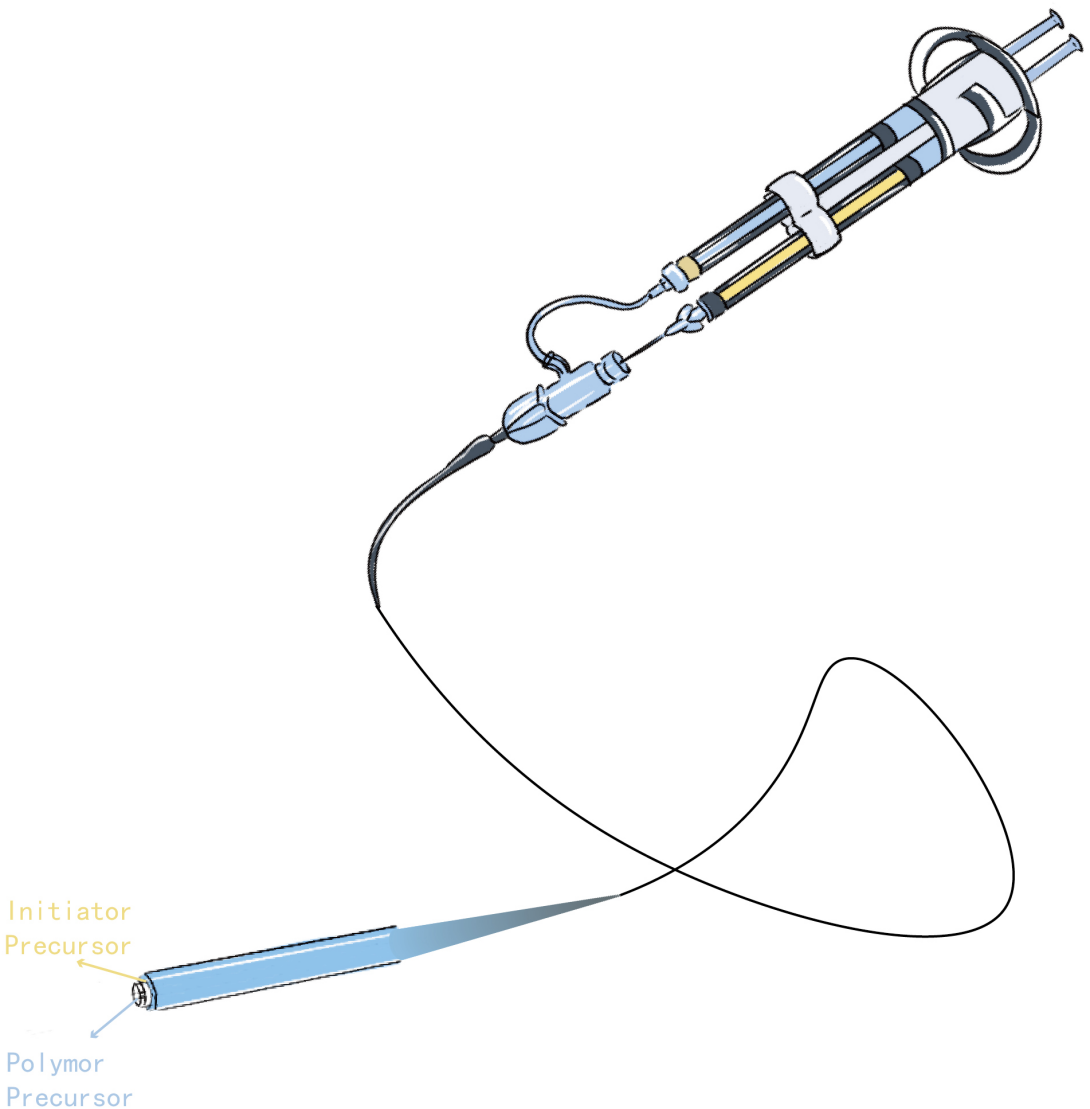
Illustration of Embrace™ Hydrogel Embolic System.

The Embrace™ Hydrogel Embolic System offers another solution by isolating the polymer precursor and initiator precursor until reaching the catheter tip. In this way, the little refluxed precursors will not reach the gelation concentration and will not accumulate at the inappropriate place^63^, ^64^ (Figure 4). Goh et al. reported the first‐in‐human of Embrace™ in 2022 involving eight patients with hepatocellular carcinoma, renal angiomyolipoma, and intrahepatic cholangiocarcinoma. All patients reached a technical success, and no recanalization of the target vessels was observed 30 days after the embolization. Besides, no serious adverse effect related to the embolic agent was observed during the 30‐ to 90‐day follow‐up.^14^

### 2.3 Interaction‐specific hydrogel

Since the 1970s, studies have explored the interaction between embolic agents and surrounding tissue, noting immune cell infiltration around long‐term embolic agents in vivo.^65^ Recent advancements in materials science, immunology, and chemoembolization have ushered in a new era of hydrogel embolic agents with physical, chemical, or biological effects.^10^ Ronald et al. observed a significant upregulation of angiogenesis factors after TAE.^66^ Tischfield et al. found that different TAE conditions altered the tumor immune.^67^ These pilot investigations underscore the potential of interaction‐specific embolic agents.

#### 2.3.1 Physical effects

**FIGURE 5:**
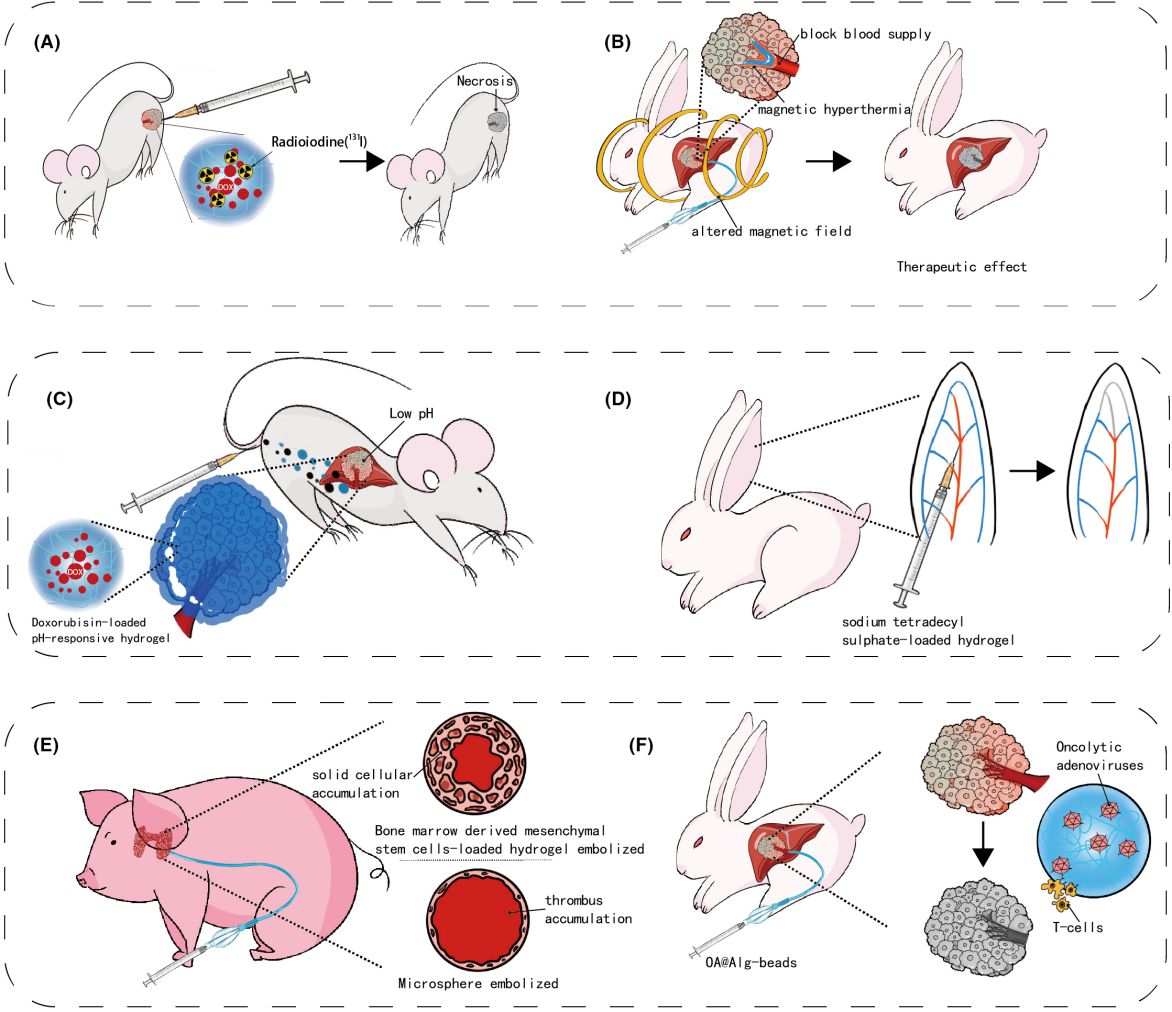
Interaction‐specific hydrogel. (A) ^131^I‐loaded hydrogel leads to tumor necrosis of rat model. (B) Illustration of a temperature‐responsive magnetic hyperthermia hydrogel assessed on VX2 liver tumor‐bearing rabbits. (C) Illustration of a doxorubicin‐loaded pH‐responsive hydrogel. (D) Illustration of a sodium tetradecyl sulfate‐loaded hydrogels leading to chemical ablation. (E) Illustration of a bone marrow‐derived mesenchymal stem cells‐loaded hydrogel, a thicker solid cellular accumulation was observed in hydrogel embolized vessels than microsphere embolized vessels. (F) Illustration of a oncolytic adenoviruses‐loaded hydrogel enhancing antitumor immune response.

Hydrogel embolic agents harness physical effects such as thermal and radioactivity. Hwan‐Jeong group has explored chitosan micro‐hydrogels, which matched the multifunctional trend of recent hydrogel embolic agents. Jeong et al. explored chitosan micro‐hydrogels, including an ^125^I‐labeled variant visible under SPECT/CT and an ^131^I‐labeled version ideal for radioembolization. In vivo experiments on a rat hepatoma model demonstrated the tumor‐suppressing effects of ^131^I‐loaded hydrogel. Additional enhancement was achieved by incorporating doxorubicin (chemical effect)^68^, ^69^, ^70^ (Figure 5A). As mentioned in the temperature‐responsive hydrogel part, Yan et al. developed a temperature‐responsive magnetic hyperthermia hydrogel, showing successful tumor artery occlusion in vivo. The temperature of this hydrogel reached 49°C under the alternating magnetic field in vitro. In vivo, embolization efficacy assessment on VX2 liver tumor‐bearing rabbits indicated successful occlusion of the tumor artery. However, the tumor volume and survival after the embolization were not measured^50^ (Figure 5B).

#### 2.3.2 Chemical effects

Most chemical effects hydrogels involve chemotherapeutics‐loaded formulations. As mentioned above, the PIB nanohydrogel, SELP, PEGDA‐nSi, HPTTG, and ^131^I‐loaded hydrogel was reported to carry doxorubicin, a widely used chemotherapeutic agent. Doxorubicin works by disrupting DNA metabolism in tumor cells and is associated with enhanced immune responses against tumors. However, systemic administration of doxorubicin can cause significant side effects, particularly cardiotoxicity. Fortunately, doxorubicin‐loaded embolic agents can deliver the drug directly to the tumor, potentially reducing the dosage required and minimizing the incidence of side effects^71^ (Figure 5C). Except for doxorubicin‐loaded SELP, which was only investigated in vitro, other doxorubicin‐loaded hydrogels demonstrated good regression of tumors in animal models.^39^, ^45^, ^57^, ^62^, ^70^ Beyond chemotherapeutics, Chabrot et al. developed a C/GP hydrogel loaded with sodium tetradecyl sulfate (STS), leading to chemical tumor ablation. Rabbit auricular artery embolization experiments demonstrated improved embolic efficacy with STS‐loaded hydrogel^72^ (Figure 5D).

#### 2.3.3 Biological effects

Hydrogel‐based embolic agents play a crucial role in biological systems, initiating intricate interactions upon administration. M.H.T. Reinges et al. reveal that hydrogel coils induce heightened neo‐endothelium and fibrous responses compared to bare coils, particularly in isolating aneurysm cavities.^73^ Joseph Touma et al. explored the application of bone marrow‐derived mesenchymal stem cells in a hyaluronic acid hydrogel for treating pig models with arteriovenous malformations. Their findings indicated an enhanced occlusion rate compared to using the hydrogel alone^74^ (Figure 5E). Peng Lv et al. loaded the calcium alginate hydrogel microsphere with oncolytic adenoviruses, leading to improved viral replication, biodistribution, reduced systemic toxicity, and heightened antitumor immune responses^75^ (Figure 5F). PuraMatrix is a self‐assembled 16 peptide consisting of repeated amino acid sequence, exhibiting low antigenicity and minimal inflammatory reactions during embolization in healthy pig models.^76^ Jingjie Hu et al. synthesized a hydrogel with decellularized cardiac extracellular matrix (ECM)‐based nanocomposite. The bio‐deprived material showed antimicrobial and pro‐regenerative effects in healthy pigs.^77^

### 2.4 Elimination‐specific hydrogel

Elimination of the embolic agents not only suggests the recanalization of the vessels but also indicates the systemic distribution of the decomposition products. However, few research about hydrogel embolic agents covered the latter. Hantao Yanga et al. reported a rapid elimination (1 h) of the synthesized hydrogel, minimizing the risk of blood clot formation.^51^ Feng Zhou et al. improved hydrogel stability by incorporating graphene oxide into poly(amidoamine) dendrimers, ensuring no deformation or migration post‐injection.^78^ It is noteworthy that certain bio‐derived hydrogels, like ECM‐based and C/GP hydrogels, exhibit relatively short recanalization times (<1 month), potentially impacting their applicability across diseases.^77^, ^79^ However, a gap remains as previous hydrogel embolic agents lack tailored designs for controlled elimination, highlighting the need for further investigation in this area.

## 3 OTHER NECESSARY PROPERTIES FOR HYDROGEL EMBOLIC AGENTS

In addition to the properties mentioned, imageability and biocompatibility are essential for hydrogel embolic agents.

Accurate visualization of the location of hydrogel during TAE procedures is crucial for precise embolization, necessitating imageability in embolic agents. Since X‐ray fluoroscopy is the most common imaging method, the hydrogel embolic agents are typically designed to be radiopaque. This is achieved by incorporating substances like iodine, gold, and tantalum into the hydrogels, with the exception of hydrogel‐coated metallic coils, which are naturally radiopaque.^32^, ^38^, ^70^ Other high‐atomic‐number materials, such as bismuth, lanthanides, and transition metal nanoparticles, also serve as contrast agents, offering diverse options for developing novel hydrogel embolic agents.^80^ While X‐ray radiography and fluoroscopy are primary imaging modalities, ultrasound and magnetic resonance imaging present promising, non‐ionizing alternatives, suggesting significant potential for developing hydrogel embolic agents suitable for these methods.^81^

Biocompatibility is also crucial. Although many animal studies have assessed hydrogel biocompatibility through blood tests and histological examinations of major organs, the complexity and variability of hydrogel embolic agents may introduce risks. For example, bio‐derived hydrogels like SELP and ECM‐based hydrogels could potentially trigger allergic reactions. Additionally, some hydrogel embolic agents have not been tested in animals, while others have only undergone short‐term safety assessments. Comprehensive long‐term safety evaluations are needed to support further clinical use of these hydrogels.

## 4 CONCLUSION AND OUTLOOK

Hydrogels represent a promising frontier in embolic agents, yet their potential extends beyond mere transportation, accumulation, interaction, and elimination. Drawing from recent advancements, multifunctionality is a trend of hydrogel embolic agents. While this manuscript discusses the in vivo procedures of hydrogel embolic agents individually, viewing them holistically is essential. The synthesized candidate hydrogels need to fulfill the requirements of the procedures. For example, during the treatment of intracranial aneurysm, the embolic agents require smooth and accurate transportation to the aneurysm site, rapid accumulation to prevent non‐target embolization, biological effects to prevent thrombosis and promote neo‐endothelium, and gradual elimination to migrate recurrence. Following these criteria, stable hydrogel or those with short sol–gel transition time are preferred. In tumor treatment, hydrogels offer unique advantages. Flowable hydrogels can effectively accumulate in peripheral vessels, facilitating complete peripheral embolization. Additionally, their robust loading capacity enables incorporation of various effect agents, including physical, chemical, and biological agents. The integration of biological effect agents holds significant promise for tumor treatment, as the role of tumor microenvironment in promoting tumor progression is well documented.^82^ Utilizing interaction agents‐loaded hydrogels to modulate the tumor microenvironment may yield optimal therapeutic outcomes.^83^

However, some controversies of embolic agents still exist. Some believe that a permanent embolization will lead to a reduced recurrence rate of malignancy. While others consider that temporary embolization will provide more chances to patients who need multiple embolotherapy. For example, in TACE therapy of hepatocellular carcinoma temporary embolization will not block the tumor‐feeding artery and will maintain more liver function when a second TACE is needed.^84^, ^85^ Therefore, in the field of hydrogel embolic agents, the property of elimination should be taken aware of. Additionally, the only shear‐thinning hydrogel embolic agent, Obsidio™ Conformable Embolic, was associated with bowel ischemia and recalled in August 2024.^86^ Despite these severe adverse events, its broad and effective embolization suggests promising potential for tumor therapy. Further research is needed to optimize the properties of hydrogel embolic agents.

## AUTHOR CONTRIBUTIONS

**Shenbo Zhang:** Data curation (equal); investigation (equal); writing – original draft (equal). **Rui Lv:** Formal analysis (equal); supervision (equal). **Zhe Zhang:** Formal analysis (equal). **Zhiwei Wang:** Conceptualization (equal); funding acquisition (equal). **Zhengyu Jin:** Project administration (equal).

## FUNDING INFORMATION

This study has received funding from the National Natural Science Foundation of China (no. 22232006, Biomolecular Condensates: Phase Separation Modulation and Functionalization) and National High‐Level Hospital Clinical Research Funding (no. 2022‐PUMCH‐B‐68).

## CONFLICT OF INTEREST STATEMENT

The authors of this manuscript declare no relationships with any companies, whose products or services may be related to the subject matter of the article.

## ETHICS STATEMENT

Approval of the research protocol by an Institutional Reviewer Board—N/A. Informed Consent—N/A. Registry and the Registration No. of the study/trial—N/A. Animal Studies—N/A.

## Data Availability

Data sharing not applicable to this article as no datasets were generated or analysed during the current study.
